# Adverse Childhood Experiences in Mexico: Prevalence and Association with Sociodemographic Variables and Health Status

**DOI:** 10.11621/pir.2023.0204

**Published:** 2023-06-30

**Authors:** Teresa Sánchez-Jáuregui, Arnoldo Téllez, Diana Almaraz, Arturo Valdez, Rogelio Hinojosa-Fernández, Hugo García-Balvaneda, Dehisy Marisol Juárez-García

**Affiliations:** Autonomous University of Nuevo Leon, Monterrey, Mexico.

**Keywords:** Adverse Childhood Experiences, mental illnesses, sexual abuse, community violence, ACE-IQ, ACEs prevalence

## Abstract

**Background:**

Adverse Childhood Experiences (ACEs) refer to a semantic field of negative childhood events that, in conjunction with insufficient personal, family, or contextual coping resources, have the potential of becoming traumatic.

**Objective:**

To assess the prevalence of Adverse Childhood Experiences (ACEs) and their association with sociodemographic variables and physical and mental illnesses in a Mexican sample.

**Design:**

A cross-sectional design was used. The sample included 917 Mexican adults who responded to the Adverse Childhood Experiences International Questionnaire (ACE-IQ). Most of the participants were female (79.3%) with an average age of 37 years, a monthly income between 500 and 2,500 USD (59.2%), had completed university education (45.6%) and were married or in a common-law marriage (53.1%). Data was collected through Google Forms, and the link to the form was shared through electronic social networks.

**Results:**

A total of 48.3% of the participants presented seven to nine types of ACEs. Among their responses, the most prevalent categories were emotional neglect (95.1%), family violence (83.3%), and emotional abuse (78.6%). A significant association was found between the number of ACEs and the mental illness diagnosis (x^2^(20) = 15.16; p<001). Women were found to report more experiences of sexual abuse (z = -6.62, *p<*. 001), whereas men reported more experiences of community violence (z= -4.27, *p* < .001) and collective violence (z = -3.94, p<.001).

**Conclusions:**

The prevalence of ACEs in the Mexican population is high. However, men and women reported differences in certain types of ACEs. It was found that people with a diagnosis and family history of mental illnesses presented a higher number of ACE categories.

## Introduction

The concept of adverse childhood experiences (ACE) has been defined as events that occur in childhood, which vary in severity and are often chronic, that occur in the child’s family or social environment and cause harm or distress and could affect the health and physical or psychological development of the child (Kalmakis & Chandler, 2014). ACEs are influenced by cultural, social, environmental, and economic factors. They cause children distress with possible cumulative effects, having repercussions on their development and physical and psychological health (Alhowaymel et al., 2021).

The dimensions included in ACEs are diverse. Some of the main ones are (1) physical, emotional, or sexual abuse; (2) emotional or physical neglect; (3) living in a family with at least one member suffering from a mental illness; (4) substance abuse; (5) incarceration or death; (6) witnessing domestic violence; and (7) parental separation or divorce (American Academy of Pediatrics, 2020).

### ACE Prevalence and Implications

Worldwide, about 60% of children have been exposed to an adverse experience and, in research done for Latin American countries, this prevalence can rise to 80% (UNICEF, 2022). In Mexico, at least 63% of children have suffered or are suffering from these events, being from rural and impoverished environments where this prevalence is higher (UNICEF, 2019).

Adversity and maltreatment negatively affect children's emotional, physical, cognitive, and social domains. There is now evidence linking ACEs to health problems among adults. The Centers for Disease Control and Prevention (CDC) reported that adults who had greater exposure to ACEs as children were more likely to have chronic health problems, depression, smoking addiction, excessive alcohol use, and socioeconomic problems compared to those who did not have adverse experiences. Furthermore, it was found that preventing ACEs could reduce the prevalence of heart disease by up to 13%, of becoming overweight or obese by 2%, and of depression by 44% among U.S. adults (CDC, 2019).

Regarding mental health, ACEs contribute to the risk of developing a wide range of psychological disorders. Among the most consistent relationships found with ACEs are anxiety disorders and depression (De et al., 2013; Heim et al., 2008; Heim et al., 2010; Li et al., 2016; Lindert et al., 2014; Liu et al., 2012; Sachs-Ericsson et al., 2017; Sareen et al., 2013; Tan &Mao, 2023); generalized anxiety disorder, social anxiety disorder, and panic disorder (Cougle et al., 2010); post-traumatic stress disorder (Cougle et al., 2010; Sachs-Ericsson et al., 2017), dysmorphic disorders (Longobardi et al., 2022); borderline personality disorder, obsessive-compulsive disorder, and schizophrenia (Battle et al., 2004); and an increased likelihood of suicide attempts and self-harm (Kappel et al., 2021).

ACEs are also positively related to risk behaviors. These include smoking, alcoholism, drug addiction, high number of sexual partners and risky sexual practices, sedentary lifestyle, and delinquency. The risk increases when one has experienced four or more ACE categories (Campbell et al., 2016; Douglas et al., 2010; Downey et al., 2017; Felitti, 1998; Ford et al., 2011; Hahm et al., 2010; Kappel et al., 2021; Tietjen et al., 2012).

The number of ACEs is also related to the presence of physical diseases. These include ischemic heart disease, cancer, chronic obstructive pulmonary disease, bronchitis, emphysema, skeletal fractures, liver disease, diabetes, and migraine (Felitti et al., 1998; Felitti et al., 2019; Li & Lacey, 2020; Merrick et al., 2019; Pierce et al., 2020; Tietjen et al., 2012). The factors mentioned above may increase the likelihood of people with ACEs dying prematurely. Brown et al. (2009) found that on average, people with six or more ACEs lived almost 20 years less than people without ACEs.

Regarding social and cognitive aspects, stressful events during childhood increase the risk of “proliferative chains’" of additional stressors. These are manifest in different aspects of life such as education, work, and socioemotional relationships. For example, these events negatively impact learning and academic performance; therefore, they affect success in educational, occupational, and socioeconomic settings in adulthood (Halfon et al., 2017; Metzler et al., 2017).

In Mexico, some ACE data have been reported in specific populations such as women and university students (Esparza-del Villar et al., 2022; Flores-Torres et al., 2020). However, these studies used instruments that did not include all the ACE dimensions considered by the World Health Organization (WHO, 2017). Therefore, the aim of the present study is to evaluate ACEs’ prevalence and their association with sociodemographic variables and the presence of physical or mental illnesses in a Mexican sample. The results of this study will be useful in generating scientific evidence on the prevalence of ACEs in the Mexican population. Primary health care is a favorable setting for the use of early adversity impact detection tools (Vega-Arce & Nuñez-Ulloa, 2017), since their identification is an opportunity to direct health care efforts to programs and specialists that can help limit the damage caused by ACEs or to help strengthen protective factors and prevent damage to health.

## Methods

### Participants

A cross-sectional design was used. The final sample consisted of 917 participants. Most of the participants were female (79.3%), with an average age of 37.8 years (SD= 12.6), with a monthly income between USD 500 and USD 2,500 (59.2%), had completed university education (45.6%), and were married or in a common-law marriage (53.1%). Additionally, 20.3%, 19.6%, and 6% reported a family history of a mental illness, physical illness, and mental illness, respectively. The most frequently reported ACE categories were emotional neglect (95.1%), family violence (83.3%), emotional abuse (78.6%), and bullying (78.3%). Approximately half of the participants (48.3%) reported that they had experienced seven to nine ACE categories *(Table 1)*.

**Table 1:** Participants’ Sociodemographic, Clinical, and Psychological Characteristics

	F (%)
Sex	
Male	190(20.7)
Female	727(79.3)
Age	
18-27	201(21.9)
28-37	299(32.6)
38-47	215(23.4)
48-57	123(13.4)
58-67	72(7.9)
67 or more	7(.8)
Monthly income (USD)	
4,000 or less	58(6.3)
4,000-9,000	211(23.0)
10,00-050,000	546(59.5)
51,000-99,000	78(8.5)
More than 100,000	24(2.6)
Schooling	
No formal education	2(.2)
Completed secondary education	9(1.0)
Truncated secondary education	1(1)
Technical education	25(2.7)
Completed high school	73(8.0)
Truncated high school education	9(1.0)
Finished university education	418(45.6)
Truncated university education	64(7.0)
Graduate degree	316(34.5)
Marital status	
Single	322(35.1)
Married/unmarried	487(53.1)
Divorced/separated	94(10.3)
Widowed	14(1.5)
Family history of mental illness	
Yes	186(20.3)
No	737(79.7)
Mental illness	
Yes	55(6.0)
No	862(94.0)
Chronic disease	
Yes	180(19.6)
No	737(80.4)
Adverse Experiences (Yes)	
Physical abuse	685(74.7)
Emotional abuse	721(78.6)
Sexual abuse	366(39.9)
Alcohol or drug abuse	272(29.7)
Incarceration	62(6.8)
Chronic mental illness	244(26.6)
Family violence	767(83.3)
Separation from parents	442(48.2)
Emotional neglect	872(95.1)
Physical neglect	384(41.9)
Bullying	718(78.3)
Community violence	698(76.1)
Collective violence	108(11.8)
Number of ACEs	
0	4(-4)
1-3	66(7.2)
4-6	321(35)
7-9	443(48.3)
More than 10	83(9.1)

*Note. F = Frequency. (%) = Percentage*.

### Measurement Instruments

#### Data Questionnaire

Sociodemographic data such as age, sex, income, and educational level were obtained. Additionally, participants were asked for any diagnoses of chronic physical and mental illnesses given by a health professional, as well as history of family diagnoses of mental illnesses.

#### Adverse Childhood Experiences International Questionnaire (ACE-IQ)

The Adverse Childhood Experiences International Questionnaire (ACE-IQ) is intended to measure exposure to adverse childhood experiences. It is designed to be administered to persons of legal age, and respondents are asked to answer questions based on their experiences during the first 18 years of their lives. It comprises a socio demographic data section and 43 items grouped into the following categories: 1) Physical Abuse, 2) Emotional Abuse, 3) Sexual Abuse, 4) Alcohol Abuse, 5) Incarceration, 6) Chronic Mental Illness, 7) Family Violence, 8) Parental Separations, 9) Emotional Neglect, 10) Physical Neglect, 11) Bullying, 12) Community Violence, and 13) Collective Violence. The response options have three formats: dichotomous for items F1-F5 (yes/no); on a 5-point Likert scale for items P1-P2 (from "never” to "always”); and on a 4-point Likert scale for all remaining items (from “never” to “many times”) (World Health Organization, 2018). This instrument has obtained reliability values greater than .80 for the overall questionnaire. The version of the instrument adapted for the Mexican population was used in the present study. It obtained an overall reliability value of .85, and .69 to .90 in the subscales.

### Procedure

Data was collected through Google Forms, and the questionnaire link was shared through electronic social networks. The participants were asked for their informed consent upon entering the questionnaire link. Here, the nature of the research and the confidential handling of the data were explained. Those who voluntarily participated were directed to the sociodemographic data and ACE-IQ forms.

### Data Analysis

The data were analyzed using SPSS 23 and descriptive data for the variables were obtained. The Chi-square test was performed to identify the association between the variables. A comparison analysis was performed between people with and without physical illnesses and those with and without mental illnesses, using the Mann-Whitney U test.

## Results

### Analysis of Associations

A significant association was found between the number of ACEs and the 38-57 years of age group (x^2^(20) = 85.45; *p <* .001) with a higher frequency in the 'Tour to seven ACEs” category. No significant association was found between ACEs and sex, schooling, or monthly income, or between the number of ACEs and physical illnesses. However, there was a significant association between the category of "four to seven ACEs” and diagnosis of a mental illness (x^2^(20) = 15.16; p < .001).

### Comparison Analysis

Regarding the prevalence in ACE types by sex, it was found that women reported more experiences of sexual abuse *(z =* -6.62, *p<* .001) and chronic mental illness of a family member *(z =* -2.13, *p <* .05), whereas men reported greater community violence (z = -4.27,p < .001) and collective violence (z= -3.94, *p<* .001).

Participants with a family history of psychiatric illness reported significantly higher frequencies of physical abuse *(z=* -2.08, p < .05), emotional abuse (z = -2.55, p<.01), a family history of violence (z = -2.75, p<.01), alcohol abuse (z=-4.51, p< .001), incarceration *(z=* -3.02, p< .001), and chronic mental illness *(z =* -11.23, p<.001).

Significant differences were also observed between people with and without physical illnesses. Specifically, those who had a physical illness had experienced more collective violence *(z= -2.26, p* < .05). Significant differences were also found between people with and without a diagnosis of a mental illness in terms of alcohol or drug abuse *(z=* -2.64, p < .01) and chronic mental illness of a family member *(z=* -4.51, p< .001). These ACE categories were more frequent among people with a diagnosis of a mental illness (Table 2).

**Table 2:** Comparison of ACE Categories

%	PA	EA	SA	AA	I	CMI	FV	PS	EN	PN	B	CV	CLV
**Sex (n)**			**			*						**	**
Female (727)	73.7	79.4	45.4	30.8	7	28.2	83.9	47.3	95	42.6	77	73	9.6
Male (190)	78.4	75.8	18.9	25.3	5.8	20.5	82.6	51.6	95.3	38.9	83.2	87.9	20
**Physical illness**												*	
Yes (180)	71.7	73.3	45	31.7	5	24.4	81.1	94.4	94.4	45.6	10	75.6	16.7
No (737)	75.4	79.9	38.7	29.2	7.2	27.1	84.3	95.3	95.3	41.0	11.4	76.3	10.6
**Mental illness**				**		**							
Yes(55)	81.8	89.1	50.9	45.5	7.3	52.7	92.7	100	100	50.9	7.3	74.5	11.7
No (862)	74.2	78.0	39.2	28.7	6.7	24.9	94.8	94.8	94.8	41.3	11.4	76.2	12.7
**Family history of mental illness**	*	*		**	*	**	**						
Yes (186)	80.6	85.5	41.9	38.7	10.2	59.1	90.3	97.3	97.3	44.6	9.7	76.9	12.4
No (731)	73.2	76.9	39.4	27.4	5.9	18.3	81.9	94.5	94.5	41.2	11.5	75.9	11.6

Note. Frequencies: physical abuse (PA), Emotional abuse (EA), Sexual abuse (SA), Alcohol abuse (AA), Incarceration (I), Chronic mental illness (CMI), Family violence (FV), Parental separation (PS), Emotional neglect (EN), Physical neglect (PN), Bullying (B), Community violence (CV), Collective violence (CLV). ** p≤.001,* p≤.05.

## Discussion

This study aims to explore ACEs’ prevalence, as well as their association with sociodemographic variables and the presence of physical and mental illnesses in a Mexican sample. An association was found between age and the number of ACEs. Specifically, the group aged 38 to 57 years reported the highest number of ACEs. However, these results differ from those found by Sonu et al. (2019), where the group aged 18 to 34 years reported the highest levels of ACEs.

In line with the studies by Choi et al. (2017) and Kidman et al. (2019), the present study also found that men had experienced greater community violence while women had experienced a higher prevalence of sexual abuse. The World Health Organization (2022) reports that one in five girls and one in 13 boys have suffered sexual abuse. Furthermore, some systematic reviews in different countries show that their prevalence rate is approaching 20% for girls and 8% for boys (Stoltenborgh et al., 2011). However, there are variations between 8-31% for girls and 3-17% for boys (Barth et al., 2013). Thus, these results are very similar to those found in the present study. Escribano et al. (2020) mentioned that there are risk factors contributing to child abuse and neglect. They highlighted gender as one of the most important in terms of sexual abuse. This is because girls are five times more likely to be abused by male figures within the family and are at a greater risk of being abused for a longer period compared to boys.

The findings discussed above are crucial, since the consequences of ACEs are different with respect to gender. Some studies even show that men are reported to have higher substance dependence and women have higher rates of chronic mental illnesses. These differences show the need to create specialized prevention programs for each gender (Almunef et al., 2017; Kappel et al., 2021).

In relation to educational level, most participants in the present study reported having completed their undergraduate and graduate university education. Moreover, 92.4% of the sample reported four or more ACEs. These results differ from the study conducted by Hardcastle (2018), where it was found that people with an incomplete university education or those who are unemployed reported four or more ACEs.

Regarding ACEs’ prevalence, the present study’s results showed that almost 100% of the sample had suffered at least one adverse experience. This result is similar to what was found in the study of Almuneef et al. (2017), who used the same instrument and a similar sample, and found that 80% of the participants reported at least one ACE. Sciolla et al. (2019) and Felitti et al. (2019) found that more than half of the sample had at least one ACE. However, these two studies used different instruments and populations. Regarding the number of ACEs experienced, the present study showed that most participants had suffered between seven and nine ACEs. It should be noted that other studies categorize the number of ACEs as “0,” “1,” “2,” “3,” and *“4 or more,”* whereas the present study categorized them as “0,” “1 to 3,” *“4* to 6,” “7 to 9” and "more than 10.” Thus, the results are similar to other studies, where most participants reported four or more ACEs (Almuneef et al., 2017; Ford et al., 2011).

In Mexico, Flores-Torres et al. (2020) pointed out that 13.7% of the sample reported four or more ACEs. This result is lower than what was found in our study, where 92.4% reported that they had suffered four or more ACEs. This may be explained by the fact that the previous study used a different instrument, with fewer categories of ACEs assessed.

Currently, child abuse and maltreatment have become alarmingly growing public health problems worldwide. They have serious consequences that result in lifelong repercussions for their victims. These problems have led to countless adults having a history of child abuse and who are at greater risk of repeating patterns of violence from one generation to the next (Benavides & Miranda, 2007).

In the case of Mexico, the situation of family violence and child abuse is alarming and requires urgent attention. In this regard, Sotelo (2014) mentions that, according to INEGI data, various studies reported that 10% of minors were victims of mistreatment and abuse in 2010.

The most prevalent types of ACEs in this sample were emotional neglect, family violence, and emotional abuse. This finding is similar to what was found in the study of Almuneef et al. (2017), where the most prevalent ACE was domestic violence. However, the present study differs from that of Felitti et al. (2019), where the most prevalent ACE was a family member’s substance abuse at home. The results are similar to what was reported in Mexico, where the highest ACE categories were physical abuse (Esparza et al., 2020) and substance abuse by a family member at home (Flores-Torres et al., 2020).

Regarding physical illness, only a few people in our study reported suffering from a chronic illness, even though there was a high prevalence of emotional and physical abuse in this research. This is different from the findings obtained in other studies, where the presence of chronic illnesses was significantly associated with emotional, physical, and drug abuse in the family (Chang et al., 2019).

However, it was found in this study that people with chronic physical illnesses had more experiences of collective violence. This is similar to what was reported by Al-Shawi and Lafta (2015), who mentioned that exposure to high levels of community-collective violence in conjunction with dysfunction-abuse in the family during childhood has serious consequences for adults’ health. Moreover, they found that these factors approximately doubled the risk of chronic illness, as compared to those with lower exposure levels. Similarly, in a study conducted in Mexico with older adult participants, it was found that greater collective violence was related to a worse health status (García-Peña et al., 2018). Several authors have reported an association between exposure to community violence and different physical conditions such as upper respiratory diseases (Wilson et al., 2005), insulin resistance, and sleep disorders among adolescents (Kliewer et al., 2019), and cardiovascular diseases among adults (Suglia et al., 2015).

The instrument used in the present study refers to secondary exposure. This is important, as the phrase exposure to violence signals a division of violence into two categories: primary exposure, which refers to a persons direct victimization; and secondaryexposure, where one observes or hears an act of violence against another person (Zimmerman & Posick, 2016). Thus, there is a division between being a victim and a witness. This is considered relevant, since being a witness is enough to affect physical and mental health (Hensel et al., 2015; Zimmerman & Kushner, 2017).

According to this study’s results, having suffered ACEs is significantly associated with a mental illness diagnosis. This finding is similar to that of other studies, suggesting that the presence of ACEs increases the likelihood of suffering from mental illnesses in adulthood (Almuneef et al., 2017; Barrera et al., 2019).

This could be explained by the fact that from birth to six years, the brain goes through its fastest period of growth and development, since this is a stage that is highly sensitive to the detrimental effects of adversity (American Academy of Pediatrics, 2020; Felitti & Anda, 2010). ACEs are early and often chronic stressors, which can lead to biological and behavioral dysregulation, which can affect functioning both physiologically and psychologically, resulting in greater sensitivity to stressors in adult life. It has been hypothesized that people exposed to early adversity have sensitive cortico-amygdala neural circuitry, which is associated with increased hypervigilance and reactivity to threatening stimuli. The amygdala acts as an initial trigger for the body’s response to stress, with mobilizing influences on the sympathetic nervous system and the hypothalamic-pituitary-adrenocortical (HPA) axis. In this way, ACEs increase physiological arousal in response to stressors, which has an effect later in life (Miller et al., 2011; Nusslock & Miller, 2016).

In our study, people who reported a family history of mental illnesses mentioned a higher frequency of ACEs. Particularly, they experienced a higher prevalence of emotional and physical abuse, family history of violence, alcohol abuse, and incarceration. Other studies have also shown that a close family member’s mental health is directly related to an increased risk of physical abuse, emotional abuse, sexual abuse, and emotional neglect during childhood (Vaithianathan et al., 2018; Wilson et al., 2015). Similarly, it has been found that the risk and frequency of ACEs in children are increased in families where either parent presented symptoms of depression, anxiety, or stress, and a history of traumatic childhood events (Lawson et al., 2020). This suggests that mental illness in either parent leads to a cycle of intergenerational maltreatment or the initiation of a new one; the transmission of physical, emotional or sexual abuse can occur in up to three generations; victims of abuse in previous generations can also perpetuate the risk factors for victimization in future generations (Badenes-Ribera et al., 2020).

## Limitations and Strengths

One limitation of the study was that the sample is extremely well educated, with 34% having a graduate degree, and the sample was self-selected via social networks. It is recommended that the study be replicated in a more heterogeneous population that includes different socioeconomic strata. Moreover, it should be carried out in different states of Mexico, to learn about the phenomenon at the national and regional levels. Lastly, future studies must explore different variables that include protective factors, such as resilience and optimism. These will help in understanding how ACEs can affect people differently according to their personality characteristics.

This study has several strengths. First, the Mexican version of the ACE-IQ was used. This instrument is recommended by the WHO to standardize the criteria by which the presence of ACEs is evaluated. Second, this study considers variables that have not been tested in other types of questionnaires, such as collective violence. Third, the present study made it possible to obtain updated prevalence rates of ACEs in Mexico, which can be compared with the results of studies from other countries. Lastly, this work can open the way for future research, such as the investigation of risk factors associated with ACEs. This can help in the development and implementation of intervention and prevention programs in vulnerable populations.

## Conclusion

This study found that ACEs’ prevalence in this sample of Mexican population is high. Most of the participants in this study presented seven to nine types of ACEs. The most prevalent ACE categories were emotional neglect, family violence, emotional abuse, bullying, community violence, and physical abuse. People with a family history of a mental illness diagnosis had a higher number of ACEs, particularly in the categories of physical abuse, emotional abuse, family history of violence, alcohol abuse, incarceration, and chronic mental illness. Females reported a higher prevalence of sexual abuse and mental illness of a family member, whereas males experienced greater community and collective violence. Furthermore, participants diagnosed with a physical illness experienced greater collective violence. Participants diagnosed with a mental illness experienced more alcohol or drug abuse and chronic mental illness by a family member.
